# The FreeD module for the Lokomat facilitates a physiological movement pattern in healthy people – a proof of concept study

**DOI:** 10.1186/s12984-019-0496-x

**Published:** 2019-02-06

**Authors:** Tabea Aurich-Schuler, Anja Gut, Rob Labruyère

**Affiliations:** 1 Rehabilitation Center Affoltern am Albis, Children’s University Hospital Zurich, Mühlebergstrasse 104, CH-8910 Affoltern am Albis, Switzerland; 2 Children’s Research Center, Children’s University Hospital Zurich, Steinwiesstrasse 75, CH-8032 Zurich, Switzerland; 3 Department of Health Sciences and Technology, ETH Zurich, Vladimir-Prelog-Weg 1-5/10, CH-8093 Zürich, Switzerland

**Keywords:** Robot-assisted gait therapy, Lateral translation, Transverse rotation, Weight shifting, Trunk movements, Pelvis, Chest, Path control, Rehabilitation, Surface electromyography

## Abstract

**Background:**

A contralateral pelvic drop, a transverse rotation and a lateral translation of the pelvis are essential features of normal human gait. These motions are often restricted in robot-assisted gait devices. The optional FreeD module of the driven gait orthosis Lokomat (Hocoma AG, Switzerland) incorporates guided lateral translation and transverse rotation of the pelvis. It consequently should support weight shifting during walking. This study aimed to investigate the influence of the FreeD module on trunk kinematics and hip and trunk muscle activity.

**Methods:**

Thirty- one healthy adults participated. A video analysis of their trunk movements was performed to investigate the lateral chest and pelvis displacement within the Lokomat (with and without FreeD), and this was compared to treadmill walking. Furthermore, surface electromyography (sEMG) signals from eight muscles were collected during walking in the Lokomat (with and without FreeD), on the treadmill, and overground. To compare the similarity of the sEMG patterns, Spearman’s correlation analyses were applied.

**Results:**

Walking with FreeD elicited a significantly higher lateral pelvis displacement and a lower lateral chest displacement (relative to the pelvis) compared to walking with a fixated pelvis. No significant differences in the sEMG patterns were found for the Lokomat conditions (with and without FreeD) when comparing it to treadmill or overground walking.

**Conclusions:**

The differences in pelvis displacement act as a proof of concept of the FreeD module. The reduction of relative lateral chest movement corresponds to a decrease in compensatory trunk movements and has its origin in allowing weight shifting through the FreeD module. Both Lokomat conditions showed very similar muscle activity patterns of the trunk and hip compared to overground and treadmill walking. This indicates that the Lokomat allows a physiological muscle activity of the trunk and hip during gait.

**Electronic supplementary material:**

The online version of this article (10.1186/s12984-019-0496-x) contains supplementary material, which is available to authorized users.

## Background

The kinematics of the pelvis play a crucial role in the control of the center of mass during human walking. Three out of six movement strategies to reduce the energy expenditure in human gait are a contralateral pelvic drop, transverse rotation and a lateral translation of the pelvis [1]. Whereas the first two are passive motions of the pelvis initiated by the swing leg, the lateral translation occurs actively. The lateral translation of the pelvis intends to shift the center of gravity over the standing leg which involves a natural valgus position of the knee and relative hip adduction during the stance phase [2]. These movements can be restricted by deformity, muscle weakness, impaired control and pain which further leads to an abnormality of the gait pattern [2].

**Fig. 1:**
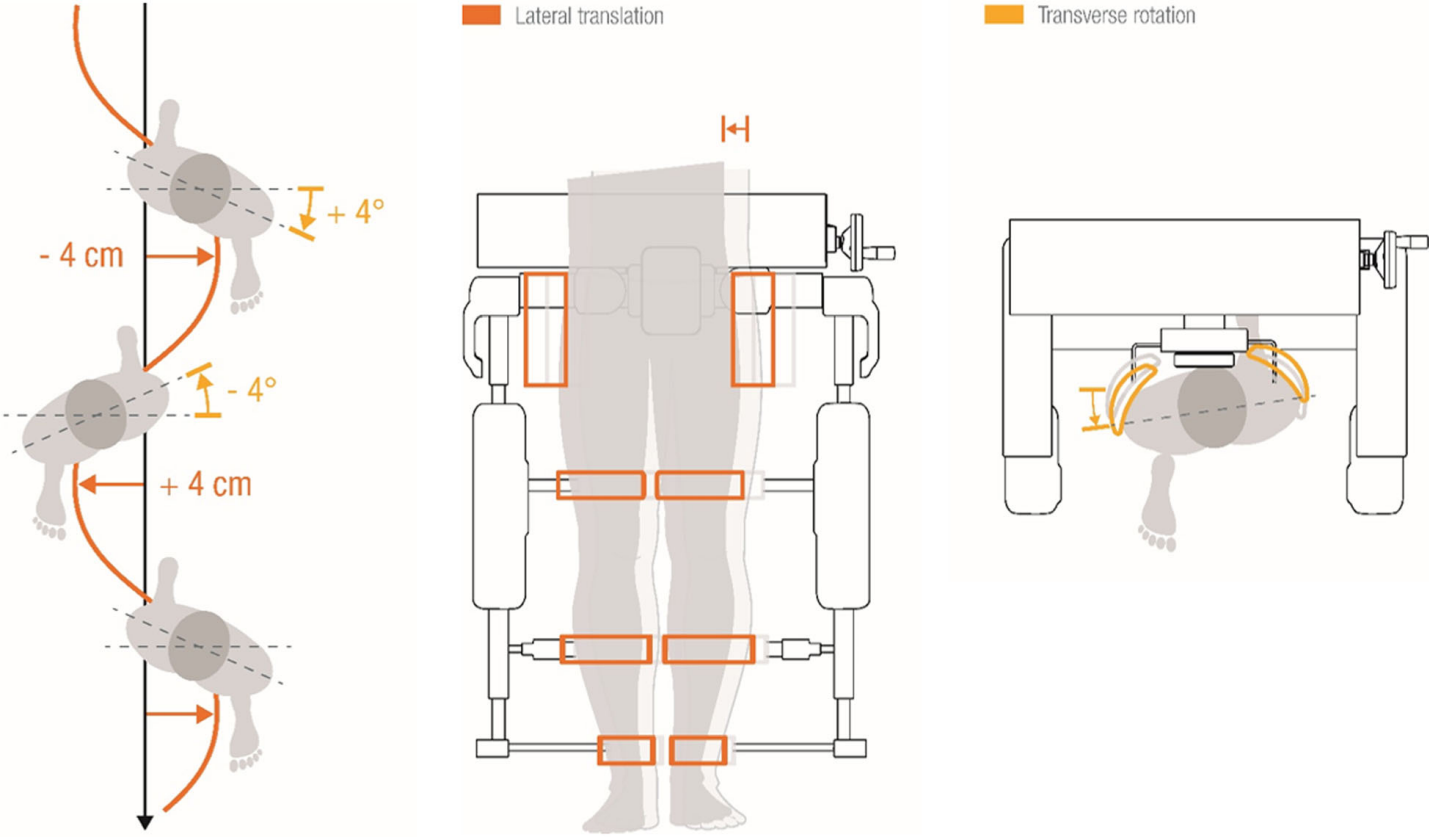
Possible pelvis movements (a combination of lateral translation and transverse rotation) of the Lokomat FreeD module (Image courtesy Hocoma AG, Volketswil, Switzerland)

In rehabilitation, in addition to conventional physical therapy, body weight supported treadmill therapy is used to train or maintain the patients’ ability to walk. It has been shown that fixating the pelvis during treadmill walking changes gait kinematics [3]. This issue translates directly to robot-assisted gait therapy, as the pelvis is usually fixated in most devices on the market. Koopman et al. mention accordingly that the fixation of the pelvis might be one of the biggest disadvantages of commonly used gait robots [4]. The pelvic restrictions are expected to influence the muscle activity patterns when walking in a robot-assisted gait therapy device on a treadmill with limited degrees of freedom to the sagittal plane [5]. For the robot-assisted gait device Lokomat (Hocoma AG, Volketswil, Switzerland), a recent commercial upgrade of the conventional technology has been released to address these limitations. The FreeD module is an optional hard- and software related control strategy with additional degrees of freedom for the pelvis and the legs which should enable weight shifting and a more normal gait pattern [6]. In contrast to the conventional Lokomat setup, the actuated FreeD module guides the pelvis to undergo lateral translation of maximally 4 cm to each side, and this translation is coupled to a transverse rotation of up to ±4° per side (Fig. 1). The robot mechanically moves the pelvis on this semi-elliptical path. The amplitude of the lateral translation, as well as the timing of the movement, can be individually adjusted, and the pelvis guidance is synchronized with the walking speed. Additionally, the leg cuffs at the thigh and upper shank can be partially released (unclenched) which makes medio-lateral displacement (of the knee and the thigh) possible. This mechanism allows the legs to follow the lateral pelvic displacement and accordingly enables a natural valgus angle at the knee during the stance phase and a weight shift to the standing leg. With this mechanism and because the patient in the FreeD is not so rigidly fixed compared to the conventional Lokomat, the manufacturer advertises that the patient can participate more actively in the lateral translation and transverse rotation. In addition, it should be possible to train balance skills which are often affected in patients with neurological disorders [3].

The discussion about the effectiveness and benefit of the Lokomat for different patient populations has been controversial for years which drove further development of the technology. The effect of the different systems must first be understood to apply the therapy in a patient-oriented way [7]. To the best of our knowledge, there exists only one study about the FreeD technology in general, and it evaluated the FreeD module in an adolescent population with neurological diagnoses [7]. The authors compared leg muscle activity patterns of FreeD and Path Control walking conditions to conventional Lokomat walking with Guidance Force [7]. Whereas Guidance Force works as a position control mode, moving the leg along a strictly defined trajectory, Path Control aims to induce a patient-cooperative behavior by allowing the user to move within a virtual tunnel around this given trajectory instead of having to stay on it. The study showed that the amount of muscular activity increased with higher kinematic freedom in patients with neuromotor disorders. Even though the authors found a less physiological pattern with the FreeD module (compared to a reference curve of typically developing children), it was not possible to draw a general conclusion about the FreeD due to the heterogeneous patient sample. Accordingly, it remains unclear whether patients’ muscle activity patterns during the FreeD condition deviated considerably from the reference pattern of healthy children due to their impairments, or because the FreeD module prevented a physiological walking pattern. Thus, it needs to be investigated, if the FreeD module allows a biomechanically normal/physiological walking pattern.

Besides that, it is expected that the FreeD module influences trunk kinematics, especially in the frontal plane. In the conventional Lokomat setup, lateral trunk flexion is increased, and lateral hip translation is decreased compared to treadmill walking in healthy subjects [8]. The increased lateral trunk flexion represents a compensatory movement, which is due to the fixation of the pelvis and the resulting lack of weight shifting. To date, there exists no literature which evaluates these trunk movements while walking with the FreeD module.

Therefore, the aim of this study was to examine the influence of guided lateral translation and transverse rotation of the pelvis in robot-assisted gait therapy on hip and trunk muscle activity patterns in healthy subjects and the following research hypotheses were formulated: (1) Lateral trunk movement in the Lokomat: The FreeD module will reduce lateral chest displacement (relative to hip) and increase absolute lateral hip displacement, and (2) sEMG patterns of hip and trunk muscles: The correlation between walking on the treadmill/overground and in the Lokomat with FreeD is higher than without.

## Methods

### Participants

Participants between 18 and 65 years of age were recruited by convenience sampling. They had to meet the following inclusion criteria; (1) no diagnosed gait abnormality, (2) a femur length of 35–47 cm (to fit in the adult Lokomat leg orthosis), (3) no contraindication to train in the Lokomat (see [9]), (4) no surgical intervention within the last three months, (5) written informed consent.

### Measurements and experimental design

Since the project did not fall under the Human Research Act, a “declaration of no objection” was issued by the Ethics Committee of the Canton Zurich.

All the measurements were performed at the Rehabilitation Center of the University Children’s Hospital Zurich in Affoltern am Albis, Switzerland.

**Fig. 2:**
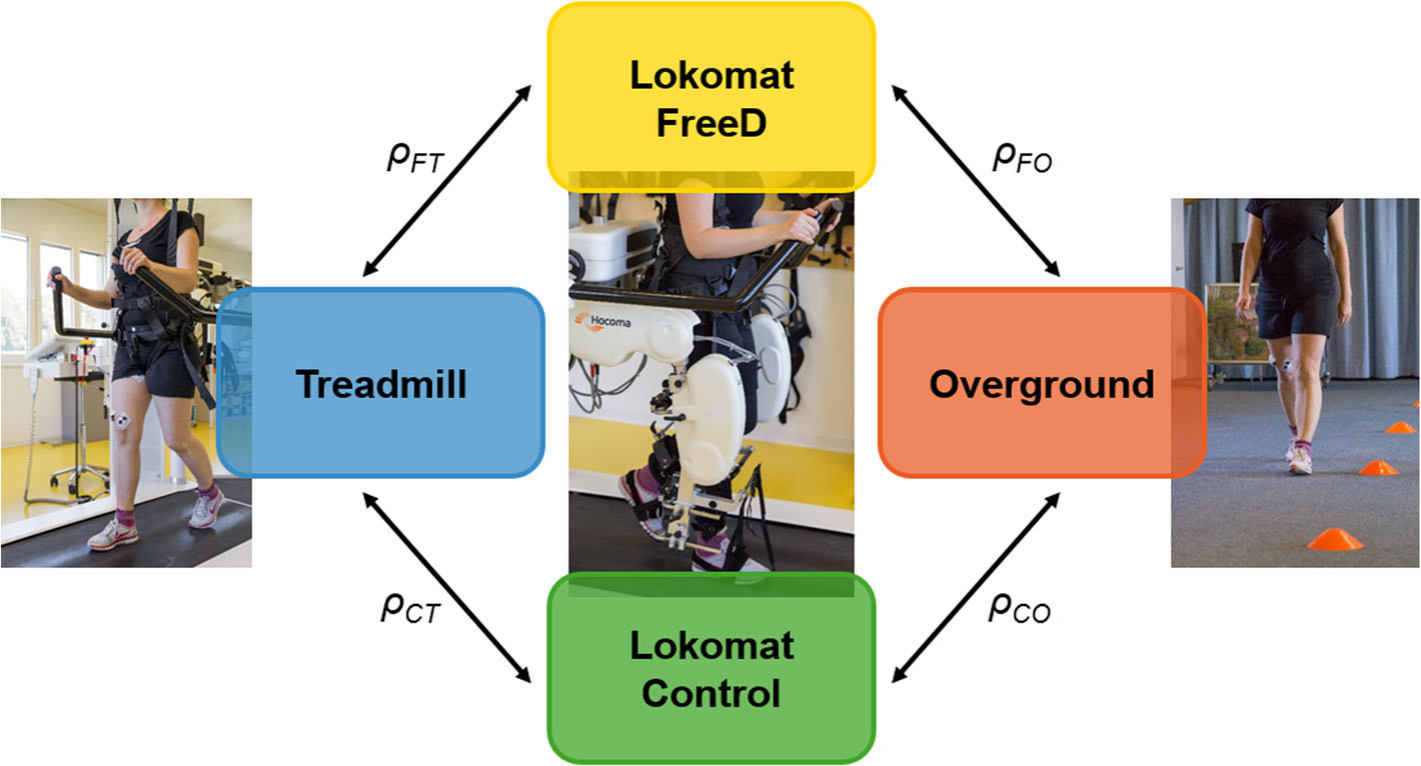
The study procedure presents the four walking conditions (Lokomat FreeD, Lokomat Control, treadmill, overground). The measurements started with the Lokomat conditions in randomized order, followed by the treadmill condition and ended with the overground condition. To compare the similarity of the sEMG patterns across the conditions, Spearman’s correlation coefficients were calculated for each participant separately which led to the new variables ρ_FT_, ρ_FO_, ρ_CT_, ρ_CO_

Figure 2 gives an overview of the four conditions applied in the study. The whole procedure lasted about two hours per person. The design of the experiment allowed an intra-subject comparison of four different walking conditions, measured in the following order: Lokomat FreeD and Lokomat Control (i/ii), Treadmill (iii), Overground (iv). The Lokomat setting was split into two randomly offered conditions: (i) Lokomat FreeD with medio-lateral movement of pelvis and legs, and (ii) the Lokomat Control condition with fixated pelvis and legs. We started with the Lokomat conditions for two reasons: First, it was necessary because of the electrodes placement (EMG transmitter boxes cannot be placed under the leg cuffs) and secondly, the walking speed selected in the Lokomat was subsequently adopted for the treadmill and the overground condition. The Lokomat and treadmill conditions lasted 10 min and the seventh minute was used for data analysis, which ensured a sufficient familiarization/acclimatization period for every condition [10]. The overground test setting lasted until sufficient valid trials were available.

The instructions given before and during the measurement were standardized and guided the participants to be active and walk as normally as possible (for details about the standardized instructions, see Additional file 1).

### Devices and outcome measures

#### Lokomat and treadmill

The Lokomat Pro (Version 6, Hocoma AG, Volketswil, Switzerland) is a driven gait orthosis used for highly repetitive and intensive gait therapy in children and adults. The device consists of an exoskeleton that can be adjusted to the patient, a dynamic body weight support system, and a treadmill that can also be used without the gait orthosis. According to the ability level of the patient, speed, Guidance Force, and body weight support can be adapted. Detailed information about the Lokomat device can be found elsewhere [11]. The Lokomat orthosis was adjusted individually to each participant according to clinical standards. For all Lokomat and treadmill conditions, the parallel bars were adjusted so that the arms, shoulders and upper body could be held in a natural, comfortable and upright position. The body weight support system was set to 0%, and the device was unloaded to compensate for its weight. This was verified by conventional body weight scales. To determine the preferred speed in the Lokomat, the treadmill speed was set to 2.5 km/h and then gradually increased to reach a speed perceived as comfortable by the participant (max. 3.2 km/h). To adjust the Lokomat individually to each participant (e.g. range of motion, step length etc.), they had to walk for a short period (max. 2 min).

We used the two new control mechanisms Path Control and FreeD motion which should allow the participant to walk with more kinematic variability and, accordingly, a more natural/physiological gait pattern [7, 12]. The control mode Path Control was applied in both Lokomat conditions, and the settings were standardized in accordance with the recommendations of Aurich (-Schuler) et al. [7]: 35% Guidance Force, 40% Support Force, and a large tunnel width (those are both settings of the Path Control mode, see [7]). For the FreeD condition, lateral pelvis translation (combined with rotation on a semi-elliptical path) was set to 2 cm to each side with a + 10% time offset. The cuffs at the thigh and upper shank were released to allow a medio-lateral shift. An explanation of the technical details of Path Control and FreeD can be found elsewhere [7].

For the treadmill condition, the Lokomat treadmill and the same body weight support system were used. Although body weight support in the treadmill condition was also set to 0%, the harness and the suspension system were attached to mimic the clinical situation.

#### Overground

The measurements ended with the overground condition. This condition was carried out on a 10-m walkway with four additional meters to accelerate and enough space to decelerate afterward. The participant was instructed not to amble, but to walk naturally. The measurement was conducted at the self-selected (preferred) Lokomat walking speed. This was enforced by setting up cones next to the walkway which had to be passed corresponding to an acoustic pace. This required a few practice trials. To control the actually performed walking speed and to detect valid trials, gait speed was measured in every trial. A minimum of two measured trials at the correct speed was required to have a total of ten strides in the video for later analysis.

#### Lateral trunk movements

A video camera (filming at 25 frames per second) was placed in front of the treadmill to assess the lateral displacement of the pelvis and the chest. For this purpose, one marker was attached to the chest, positioned on the upper end of the sternum, and one marker on a tight belt around the pelvis. Additionally, a reference stripe with a length of 10 cm was stuck to the chest to calibrate distances. Using the free two-dimensional video analysis software Kinovea (open source General Public License v2, version 0.8.26, http://www.kinovea.org), the position of the markers in the frontal plane over time could be extracted offline. To reduce the effect of the markers moving towards/away from the camera, the participants were instructed to hold the position on the treadmill and keep their hands on the parallel bars during the measurement. The recording of the trunk movements could only be done during the Lokomat and treadmill condition, but not during overground walking condition.

#### Surface electromyography (sEMG)

Muscle activity was measured on the right body side using sEMG electrodes attached to the trunk (M.erector spinae, M.obliquus externus abdominis, M.rectus abdominis), hip (M.gluteus medius, M.gluteus maximus, M.tensor fascia latae), and thigh (Adductors, M.vastus medialis). The preparations for the sEMG measurement were always performed by the same person. To ensure good signal conduction, the skin was shaved, cleaned, and rubbed with an abrasive gel. Then, eight self-adhesive Ag/AgCl dual snap electrodes (10 mm diameter each and 20 mm inter-electrode distance, Noraxon Inc., Scottsdale, USA) were placed according to the SENIAM recommendations (legs [13]) and Boccia et al. (trunk [14]). The quality of the sEMG signals was permanently observed by an assisting person and sources of movement artifacts (due to interference with the harness, contacting cables, or due to dropping electrodes) were eliminated immediately.

The sEMG signal was recorded (sampling rate of 1500 Hz) with the wireless TeleMyoDTS system (Noraxon Inc., Scottsdale, USA, CMRR > 100 dB, first-order highpass hardware filter of 10 Hz) and the corresponding software applications MyoResearchXP and myoMUSCLE (Noraxon Inc., Scottsdale, USA). The system was time-synchronized with a webcam (filming at 30 frames per second) that was positioned laterally to the measured leg to trigger the gait cycle events in the sEMG software.

### Data processing

#### Lateral trunk movements

With the video analysis software Kinovea, we extracted the spatial coordinates of the pelvis and chest marker over time. In MATLAB, the mean horizontal position was defined as the zero position for the marker movement. First, all the absolute peak displacement values were identified for the pelvis marker. To take into account possible lateral shifts of the participant on the treadmill, a peak value of the pelvic marker in the treadmill condition was defined as half the distance to the consecutive opposite peak. The median of all peak values was then used for further calculations to reduce the influence of above average peaks resulting from a lateral shift on the treadmill. The resulting range of motion (ROM) in the horizontal direction of the hip marker corresponded to the median of the peak-to-peak amplitude (sum of displacements to both sides). Furthermore, the horizontal position of the pelvis marker was subtracted from the chest at each time point, and the same procedure as for the pelvis was applied to the resulting variable. Accordingly, the outcome measure for chest movement was the lateral ROM relative to the pelvis marker.

#### sEMG

First, data processing of the sEMG signal was performed directly in the Noraxon software. Gait events were triggered manually based on the video recordings. Movement artifacts were observable, especially in muscles under the harness and pelvis orthosis. Therefore, a 20 Hz Butterworth high-pass filter was applied to the raw signal to eliminate these artifacts [15]. Additionally, the signals were rectified and smoothed by Root Mean Square (RMS) with a time window of 100 ms. Afterward, the sEMG data of ten strides [16] were exported to MATLAB (R2016a, the MathWorks Inc., Natick MA, USA) for further processing. Since the precise duration of the gait cycle intervals (stance- and swing phase) varies between individuals and steps [2], the time point of the toe off for data analysis was artificially set to 60% to enable a comparison of strides across the different walking settings and conditions [2]. Therefore, the individual stance phase (according to the real heel strike and toe off) of each stride was resampled to 600 and the swing phase to 400 data points, so that each stride consisted of 1000 data points. Then, an average sEMG envelope over ten strides was calculated per subject and muscle.

**Fig. 3:**
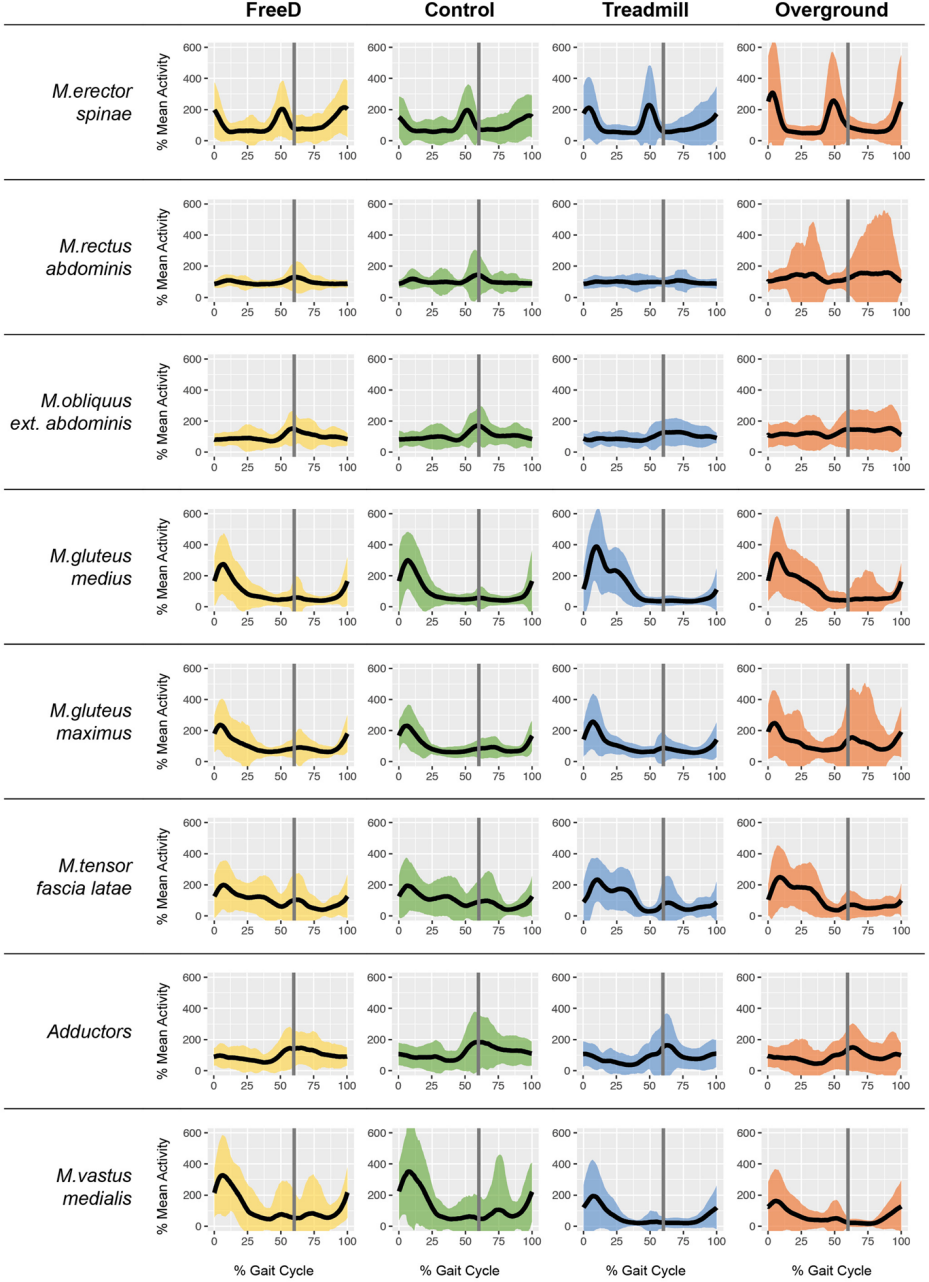
Overview of all averaged sEMG activity normalized to the mean amplitude of Lokomat and treadmill walking. The grey line at 60% of the gait cycle indicates the normalized toe-off. The 95% confidence interval is shown by colored areas. Mean walking speed for all conditions was 3.0 km/h

For the visual display (Fig. 3), the inter-subject sEMG variability was reduced by normalization of each participant’s individual sEMG amplitude to the mean amplitude of his Lokomat and treadmill sEMG activity per muscle [17]. Afterward, the activity pattern of each muscle was averaged over all subjects.

### Data analysis and statistics

The statistics were done with RStudio (RStudio Team (2015). RStudio: Integrated Development for R (Version: 0.99.903). RStudio, Inc., Boston, MA, USA, URL: http://www.rstudio.com). First, data were checked for normality with the Shapiro-Wilk test, considering skewness and kurtosis, as well as visual inspections of Q-Q-plots and histograms. As expected, most of the data were not normally distributed. Consequently, non-parametric tests (Wilcoxon signed-rank test, Spearman’s correlation coefficient) were used for subsequent analyses.

To compare the similarity of the sEMG patterns across the conditions (Lokomat FreeD vs. treadmill, Lokomat Control vs. treadmill, Lokomat FreeD vs. overground and Lokomat Control vs. overground), Spearman’s correlation coefficients were calculated for each participant separately. Correlation analyses are sensitive to similarities in shape of sEMG patterns (similar shape will yield a high correlation), but not so sensitive to similarities in sEMG amplitudes (even if there are rather large differences in amplitude, the correlation will still be high, if the pattern is the same). Differences between these new variables (correlation coefficients ρ_FT,_ ρ_CT,_ ρ_FO,_ and ρ_CO,_ see Fig. 2) and pelvis/chest lateral displacement of the Lokomat conditions were further analyzed using a two-sided Wilcoxon signed-rank test. Post-hoc corrections for multiple testing were done by applying the False Discovery Rate (FDR) [18]. The correlations of the sEMG comparisons were interpreted as follows (adopted from [19]): r < 0.20, “very weak”; 0.20–0.39, “weak”; 0.40–0.59, “moderate”; 0.60–0.79, “strong” and 0.80–1.00 “very strong relationship”.

A 95% confidence interval (CI) and an alpha value of 0.05 were used for all calculations. The effect size (r) of the Wilcoxon signed-rank test was calculated, and an effect size of r = 0.1 was interpreted as small, r = 0.3 as medium, and r = 0.5 as large [20, 21].

## Results

### Participants

In total, 31 healthy adults (7 men, 24 women) participated in the study. They chose a mean preferred (self-selected) walking speed of 3.0 km/h in the Lokomat. The main characteristics of the participants are summarized in Table 1.

**Table 1:** Characteristics of the participants

	Body mass (kg)	Body height (cm)	Age (years)	Walking Speed (km/h)
Mean	65.9	170	31.4	3.0
SD	9.9	7	9.8	0.1
Range	49–94	157–188	18.0–56.8	2.7–3.2

Abbreviation: *SD* Standard deviation

### Lateral trunk movements

The analysis of the pelvis and chest displacement during the Lokomat Control condition with fixated pelvis revealed the expected anticyclical behavior (“compensatory movements”, Fig. 3, top left panel). Compared to that, both displacement curves were synchronized during walking with FreeD (Fig. 3, top middle panel). The FreeD patterns thereby were very similar to those during treadmill walking (Fig. 3, top right panel). But in contrast to the treadmill condition, with FreeD the maximal amplitude of the hip curve was still lower than that of the chest curve. The bottom panels of Fig. 3 show the median ROM (peak-to-peak displacement) of the pelvis marker for each participant in each condition (except overground walking). During the Lokomat Control condition, the median ROM over all participants was 1.79 cm (interquartile range (IQR) = 0.69). This significantly increased to 2.58 cm (IQR = 0.74, *p* < 0.001, effect size = 0.75) for the Lokomat FreeD condition. In comparison, the median ROM during treadmill walking was 4.75 cm (IQR = 1.47). The median ROM of the chest marker relative to the pelvis marker was 4.03 cm (IQR = 1.95) for the Lokomat Control condition and that significantly decreased to 2.27 cm (IQR = 1.80) in the Lokomat FreeD condition (*p* < 0.001, effect size = 0.95) The relative chest ROM of treadmill walking had a median of − 2.35 cm (IQR = 1.20), and it is negative since the maximal hip marker excursion was generally larger than the chest marker excursion.

### sEMG

**Fig. 4:**
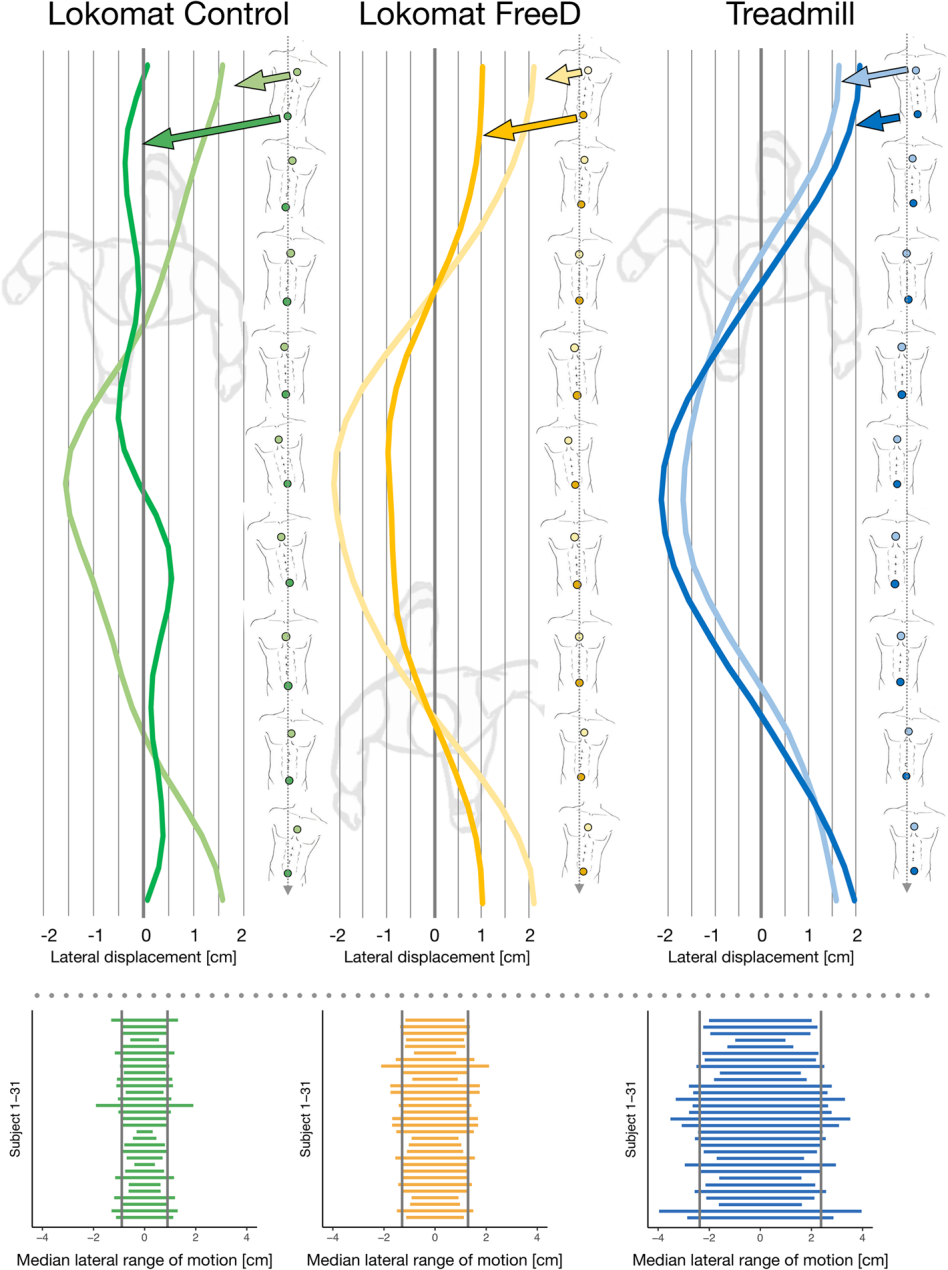
Mean lateral displacement of the chest and pelvic marker for one stride over time from a bird’s eye view (upper panel, Control in green, FreeD in orange, and Treadmill in blue, average of 20 strides). To the right of those graphs, the according upper body movement is depicted. The lower panel shows the median lateral range of motion (peak-to-peak displacement) of the pelvic marker of each subject. Thereby, the grey vertical lines indicate the median values of the group

The averaged sEMG activity of all measured muscles is shown in Fig. 4. The overground walking sEMG data of three participants had to be excluded due to video synchronization problems.

Table 2 presents the correlation statistics of the sEMG patterns of each muscle between the conditions outlined in Fig. 2. The Wilcoxon signed-rank tests revealed that neither of the two Lokomat conditions showed more similar sEMG patterns to treadmill or overground walking than the other. Nevertheless, the percentages of positive/negative differences and the medium effect sizes indicate that the muscle activity patterns during FreeD walking have a tendency to be slightly more similar to treadmill and overground walking compared to the Lokomat Control condition (except for the M.vastus medialis and M.erector spinae).

**Table 2:** Distribution (median, IQR) and statistical analysis of the intra-subject Spearman’s correlation coefficients (ρ) between the conditions

		Median	IQR	pos./neg. Differences [%]	Median of the Difference	Wilcoxon (*p*-value)	FDR- corrected p-value	Effect Size
*M.erector spinae*	*ρ*_*FT*_	0.486	0.309	45.2 / 54.8	−0.065	0.318	0.462	0.186
	*ρ*_*CT*_	0.601	0.275					
	*ρ*_*FO*_	0.449	0.363	57.1 / 42.9	0.016	0.552	0.631	0.110
	*ρ*_*CO*_	0.560	0.302					
*M.rectus abdominis*	*ρ*_*FT*_	0.236	0.353	61.3 / 38.7	0.142	**0.046**	0.243	**0.371**
	*ρ*_*CT*_	0.148	0.384					
	*ρ*_*FO*_	0.191	0.416	57.1 / 42.9	0.125	0.236	0.419	0.220
	*ρ*_*CO*_	0.067	0.407					
*M.obliquus ext. abdominis*	*ρ*_*FT*_	0.450	0.582	80.6 / 19.4	0.093	**0.010**	0.134	**0.480**
	*ρ*_*CT*_	0.297	0.462					
	*ρ*_*FO*_	0.293	0.365	57.1 / 42.9	0.027	0.646	0.689	0.085
	*ρ*_*CO*_	0.363	0.305					
*M.gluteus medius*	*ρ*_*FT*_	0.739	0.365	58.1 / 41.9	0.031	0.421	0.562	0.149
	*ρ*_*CT*_	0.683	0.325					
	*ρ*_*FO*_	0.716	0.405	75.0 / 25.0	0.047	0.066	0.265	0.341
	*ρ*_*CO*_	0.631	0.386					
*M.gluteus maximus*	*ρ*_*FT*_	0.585	0.354	54.8 / 45.2	0.010	0.809	0.809	0.045
	*ρ*_*CT*_	0.636	0.268					
	*ρ*_*FO*_	0.668	0.353	67.9 / 32.1	0.041	**0.017**	0.134	**0.444**
	*ρ*_*CO*_	0.576	0.318					
*M.tensor fascia latae*	*ρ*_*FT*_	0.667	0.245	61.3 / 38.7	0.023	0.195	0.419	0.240
	*ρ*_*CT*_	0.643	0.326					
	*ρ*_*FO*_	0.604	0.248	64.3 / 35.7	0.037	0.126	0.335	0.284
	*ρ*_*CO*_	0.575	0.277					
*Adductors*	*ρ*_*FT*_	0.584	0.186	77.4 / 22.6	0.081	0.090	0.289	0.315
	*ρ*_*CT*_	0.512	0.263					
	*ρ*_*FO*_	0.605	0.370	64.3 / 35.7	0.035	0.227	0.419	0.224
	*ρ*_*CO*_	0.514	0.423					
*M.vastus medialis*	*ρ*_*FT*_	0.729	0.453	41.9 / 58.1	−0.010	0.318	0.462	0.186
	*ρ*_*CT*_	0.740	0.406					
	*ρ*_*FO*_	0.673	0.280	60.7 / 39.3	0.009	0.493	0.607	0.127
	*ρ*_*CO*_	0.668	0.377					

Statistically significant *p*-values before FDR correction and corresponding effect sizes are in bold. The positive/negative differences [%] represent the percentage of participants who revealed a positive difference/negative difference between ρ_FT_ - ρ_CT_ or ρ_FO_ - ρ_CO_. Abbreviations: *IQR* Interquartile range, *FDR* False Discovery Rate, *F* FreeD, *C* Control, *T* Treadmill, *O* Overground; new variables ρ_FT_, ρ_FO_, ρ_CT_, ρ_CO_ correspond to Fig. 2

**Fig. 5:**
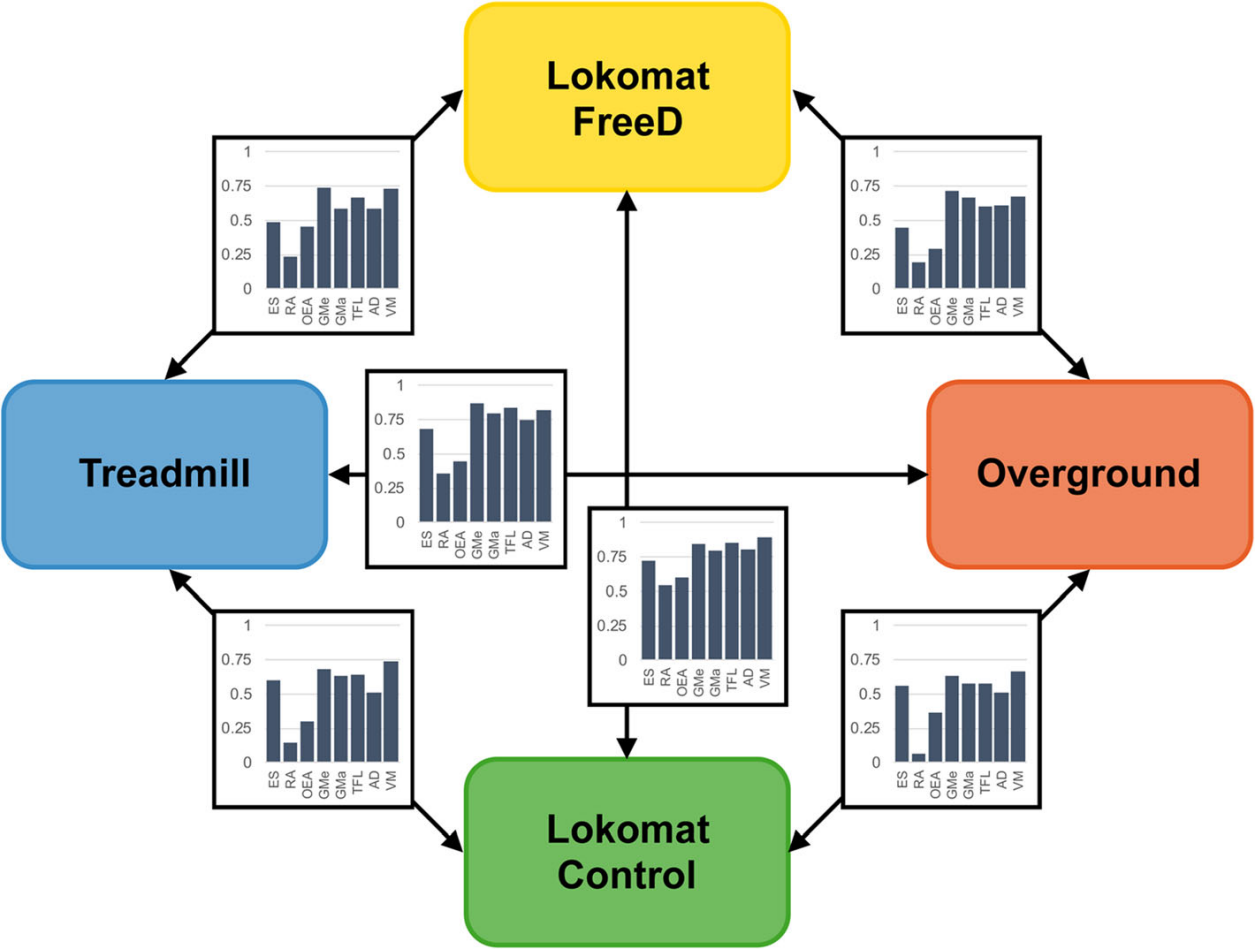
Spearman’s correlation coefficients between muscle activity patterns of all eight muscles for all walking conditions. The y-axes of the bar charts show the size of the Spearman correlation coefficient and the x-axes show the single muscles. Abbreviations: ES = M.erector spinae, RA = M.rectus abdominis, OEA = M.obliquus ext.abdominis, GMe = M.gluteus medius, GMa = M.gluteus maximus, TFL = M.tensor fascia latae, AD = Adductors, VM = M.vastus medialis

A graphical representation of the Spearman’s correlation coefficients between the sEMG patterns for all performed conditions is shown in Fig. 5. From a qualitative perspective, there appears to be a consistent pattern of correlations with more similar (high correlations) activity patterns in the leg muscles and less similar (small correlations) in the abdominal muscles.

## Discussion

This study was conducted to evaluate the influences of guided lateral translation and transverse rotation of the pelvis with the Lokomat FreeD on hip and trunk muscle activity and movement patterns in healthy subjects.

### Lateral trunk movements in the Lokomat with and without FreeD

We assumed that the FreeD module would reduce lateral chest displacement relative to the pelvis and increase the absolute lateral pelvis displacement. Our data confirm these hypotheses. For both kinematic measures of interest, the effect sizes were large. These results indicate that the FreeD can enable a more natural trunk movement (compared to the Lokomat Control condition) which can be seen as a first proof of concept of the FreeD module.

Although the pelvis ROM was larger with FreeD, there was also a certain amount of kinematic freedom in the Lokomat Control condition despite the fixation in the Lokomat. This observation is in line with previous Lokomat studies [8, 22] and can be explained by the flexibility of the attachment between the harness/cuffs and the Lokomat. Furthermore, it should also be mentioned that our Lokomat Control condition was also performed in the Lokomat FreeD exoskeleton, simply without activating the pelvic movement and opening the cuffs. Thus, the test person had a little more freedom than it would be possible in the conventional Lokomat without the optional FreeD module.

A fixated pelvis (Lokomat Control) usually leads to compensatory movements of the upper body to keep the center of mass over the standing leg. This is also reported by Swinnen et al. [8] who measured an increase in lateral flexion of the trunk during conventional Lokomat walking compared to treadmill walking. Our results showed a decrease in chest displacement relative to the pelvis during the FreeD condition which indicates a reduction of these compensatory movements. Thus, the FreeD condition was closer to treadmill walking even though the lateral chest displacement was still higher than the hip displacement.

### Comparison of hip and trunk sEMG patterns: Treadmill & Lokomat FreeD (ρ_FT_) versus treadmill & Lokomat control (ρ_CT_); and overground & Lokomat FreeD (ρ_FO_) versus overground & Lokomat control (ρ_CO_)

We expected that the correlations between muscle activity patterns during walking on the treadmill/overground and in the Lokomat with FreeD would be higher than the correlation between treadmill/overground and the Lokomat Control condition: ρ_FT_ > ρ_CT_ and ρ_FO_ > ρ_CO_. However, no adaptions of the sEMG patterns could be found in the measured muscles when comparing walking with and without FreeD to treadmill/overground walking (Table 2). This is also reflected in the visual representation (Fig. 4) which shows very similar patterns between both Lokomat conditions. Nevertheless, three muscles showed medium effect sizes regarding a trend towards higher similarity of FreeD walking versus treadmill (M.rectus abdominis and M.obliquus ext.abdominis) and of FreeD walking versus overground (M.gluteus maximus) compared to the control correlation.

Visually, the sEMG patterns of the M.erector spinae, M.gluteus medius, M.gluteus maximus, and the adductors were similar to the findings of Hidler and Wall [5] or Van Kammen et al. [23] (Fig. 4). For the abdominal muscles, the M.tensor fascia latae, and the M.vastus medialis, there are currently no reference data for Lokomat walking available. Also for treadmill walking, the sEMG signals of the hip and thigh muscles did not deviate from earlier experiments [5, 23, 24]. The patterns of the trunk muscles M.rectus abdominis and M.obliquus ext. abdominis during treadmill walking were in line with Anders et al. [25], even though, in our results, the M.obliquus ext. abdominis showed an activity peak at toe off during both Lokomat walking conditions that could have its origin in the kinematic restriction of the pelvis or trunk. The M.vastus medialis was the only muscle which showed noticeable differences in sEMG amplitude across the conditions. During walking, the muscle acts as major knee extensor during the loading response until early midstance. In the Lokomat conditions, the muscle was more active during mid-swing, whereas it was rather passive during treadmill or overground walking. The reason for the increased activity in the Lokomat might be that the participants pressed their lower leg against the orthosis to increase the step length. On the treadmill and overground, however, the sEMG activity was probably much lower due to the slow walking speed [26].

Overall, the sEMG results of both Lokomat conditions (FreeD and Control) showed very similar and physiological activity patterns which is in line with Aurich (-Schuler) et al. [27] who generally reported physiological muscle activity patterns when walking in the Lokomat with the conventional control mode Guidance Force. Accordingly, the FreeD module does not prevent physiological muscle activity patterns as long as participants are able to generate them. This serves as a second proof of concept that the FreeD module generally works. Furthermore, it seems to allay the concerns of Aurich (-Schuler) et al. [7], where the sEMG patterns of adolescents with neurological gait disorders were less physiological when walking with the FreeD module. However, in their study, the settings of the FreeD were selected to enable the highest possible kinematic freedom (e.g. minimal Guidance Force) which might have provided too little support for the participating patients with neurological gait disorders. This endorses the opinion to use the FreeD only in patients who are able to generate a basic, physiological gait pattern by themselves (with the necessary support from the therapist).

As mentioned above, the previously known differences in muscle activity patterns between Lokomat and treadmill could not be reduced by the additional degrees of freedom provided to the pelvis and legs during the FreeD condition. An apparent difference in both Lokomat conditions was the absence of the second peak of the M.gluteus medius seen in treadmill walking at approximately 25% of the gait cycle (Fig. 4). As suggested by Semciw et al. [28], this peak in the sEMG signal is related to the contralateral forward rotation of the pelvis. Consequently, it can be assumed that the FreeD still does not facilitate a self-initiated pelvic movement, which makes sense considering the fact that the pelvis is being moved by the robot. However, it has to be taken into account that the participants in this study were healthy adults and that the FreeD module could have different effects on patients’ walking pattern. Hsu et al., for instance, found an increase in muscular activity in the affected leg when applying a mediolateral force to the pelvis in hemiparetic patients [29]. And Wu et al. suggested that applying an assistive force to the pelvis facilitates weight shifting, leads to an additional challenge in balance control, and consequently results in better motor control of abductor and adductor muscles [30]. Both might be responsible for an improvement in gait function in children with cerebral palsy.

In this study, we found adaptations in trunk kinematics elicited by the FreeD module. However, this did not result in differences in muscle activity patterns between the Lokomat conditions. There exist several possible explanations for this discrepancy. On the one hand, the differences between the two Lokomat conditions may not have been large enough to cause a change in muscle activity in healthy individuals or the healthy volunteers tried to keep the muscular gait pattern as natural as possible by changing their kinematics. On the other hand, the pelvic movement in the FreeD is actuated and not necessarily voluntary initiated, which could have limited a change in muscle activity. Accordingly, further studies should investigate whether the robotic actuation of the FreeD module actually hinders an actively induced weight shifting in the Lokomat and whether a passive module (comparable to the rails on the cuffs) would not be preferable.

### Limitations

The settings of the FreeD motion were the same for all participants according to clinical practice and experience from a previous study [7]. It is unclear whether different or even individually adjusted parameters would have influenced the results. Further studies should investigate the optimal settings of the FreeD module for an individualized approach. Additionally, although the body weight support was set to 0%, the gait pattern on the treadmill might still have been affected by wearing the harness and the leg straps. However, since we wanted to work close to clinical application, we decided to keep the settings the same for Lokomat and treadmill walking. For overground walking, obviously, the harness was not used.

During the sEMG data acquisition, technical problems led to a desynchronization of the video that was recorded to trigger the gait events in the sEMG signal. All recordings were checked manually, and in the end, three data files had to be rejected because the problem could not be eliminated.

The method applied to measure the trunk movements is not a validated instrument for kinematic analyses, although there are indications that the kinovea software is a valid and reliable tool to obtain distance dimensions [31]. It has to be mentioned that we determined the translation from a two-dimensional frontal image, whereas the actual translation of the pelvis is even larger since the hip undergoes a transverse rotation in addition to the translation guided by the FreeD module. In the treadmill condition, the position of the participant relative to the camera could not be kept exactly the same which could have led to minimal changes in the amplitudes of the marker movement. Moreover, the marker placement did not align with exact anatomical landmarks. Therefore, the results should be interpreted carefully. Nevertheless, the outcomes gave us a first idea of meaningful kinematic effects and can hopefully be validated in the future with state of the art kinematic measurements.

Although our results provide a proof of concept for the FreeD, they cannot simply be generalized to patients, because healthy subjects may be able to show a normal gait pattern even under difficult circumstances. Therefore, further studies with patients are needed to clarify this issue.

## Conclusion

This study analyzed the trunk movements during Lokomat walking with and without the FreeD module and during treadmill walking. Furthermore, it compared the sEMG patterns of hip and trunk muscles while walking in the Lokomat with and without FreeD to those of walking on the treadmill and overground. The FreeD did have an influence on hip and trunk kinematics in the frontal plane. The reduction of relative lateral chest movement corresponds to a decrease in compensatory trunk movements and has its origin in allowing weight shifting through the FreeD module.

The performed physiological muscle activity patterns and the changes in trunk kinematics correspond to a proof of concept of the FreeD module.

Both Lokomat conditions showed very similar muscle activity patterns of the trunk and hip compared to overground and treadmill walking. This indicates that the Lokomat allows a physiological muscle activity of the trunk and hip during gait, irrespective of the use of the FreeD.

## Additional files

Additional file 1: Standardized test instructions. (DOCX 16 kb)

Additional file 2: STROBE Statement checklist. (DOCX 21 kb)

Additional file 3: Source data. (ZIP 16134 kb)

## Abbreviations

CI: Confidence Interval
FDR: False Discovery Rate
IQR: Interquartile Range
ROM: Range of motion
sEMG: Surface electromyography
