# Correction to: The FreeD module for the Lokomat facilitates a physiological movement pattern in healthy people – a proof of concept study

**DOI:** 10.1186/s12984-019-0541-9

**Published:** 2019-06-11

**Authors:** Tabea Aurich-Schuler, Anja Gut, Rob Labruyère

**Affiliations:** 1 Rehabilitation Center Affoltern am Albis, Children’s University Hospital Zurich, Mühlebergstrasse 104, CH-8910 Affoltern am Albis, Switzerland; 2 Children’s Research Center, Children’s University Hospital Zurich, Steinwiesstrasse 75, CH-8032 Zurich, Switzerland; 3 Department of Health Sciences and Technology, ETH Zurich, Vladimir-Prelog-Weg 1-5/10, CH-8093 Zürich, Switzerland

**Correction to: J Neuroeng Rehabil**

**https://doi.org/10.1186/s12984-019-0496-x**

The original article [1] contains an error whereby the legends of Figs. 3 and 4 are erroneously swapped. As such, the correct configuration of these legends can be seen in the same figures below instead.

**Fig. 3:**
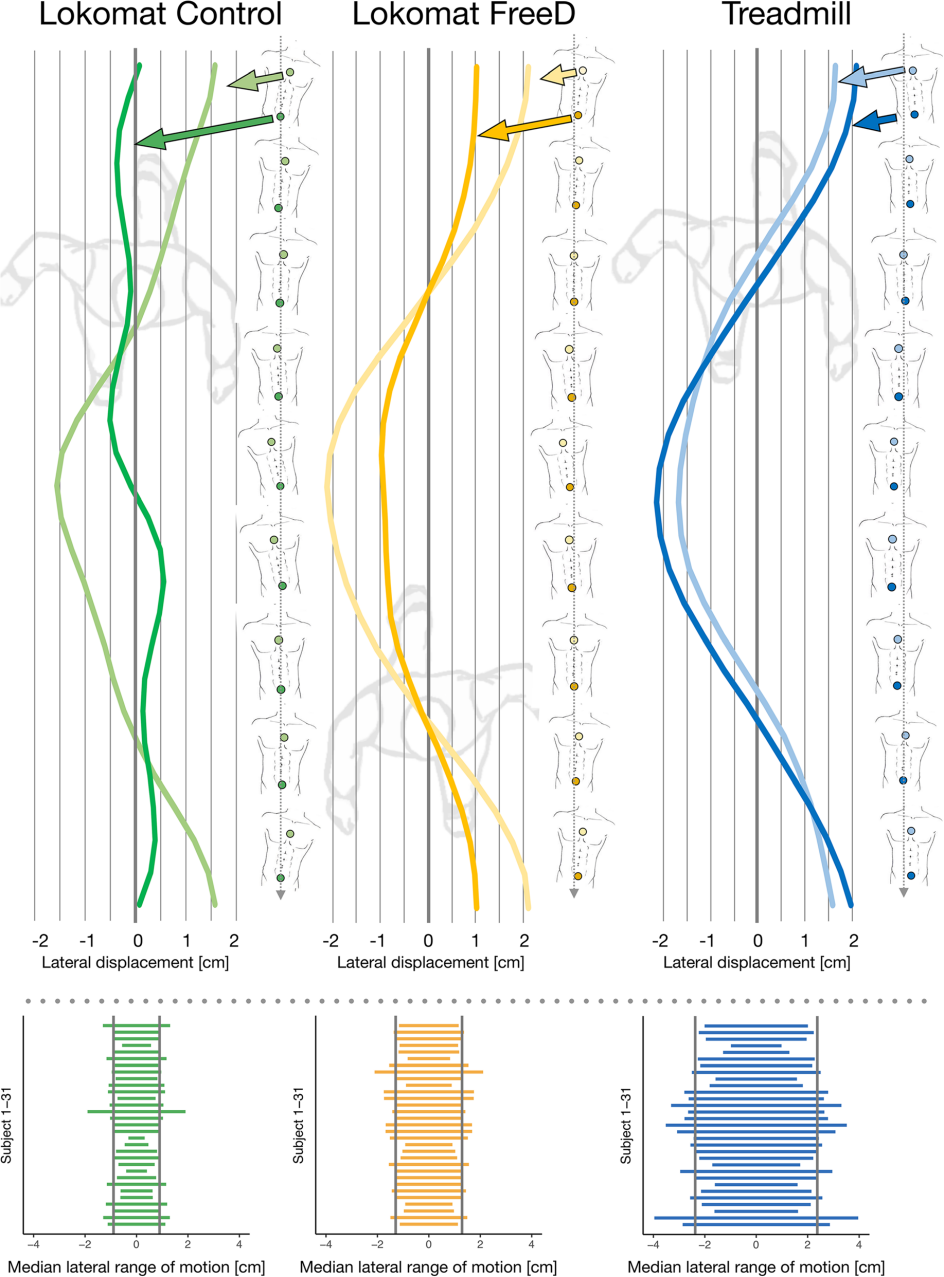
Mean lateral displacement of the chest and pelvic marker for one stride over time from a bird’s eye view (upper panel, Control in green, FreeD in orange, and Treadmill in blue, average of 20 strides). To the right of those graphs, the according upper body movement is depicted. The lower panel shows the median lateral range of motion (peak-to-peak displacement) of the pelvic marker of each subject. Thereby, the grey vertical lines indicate the median values of the group

**Fig. 4:**
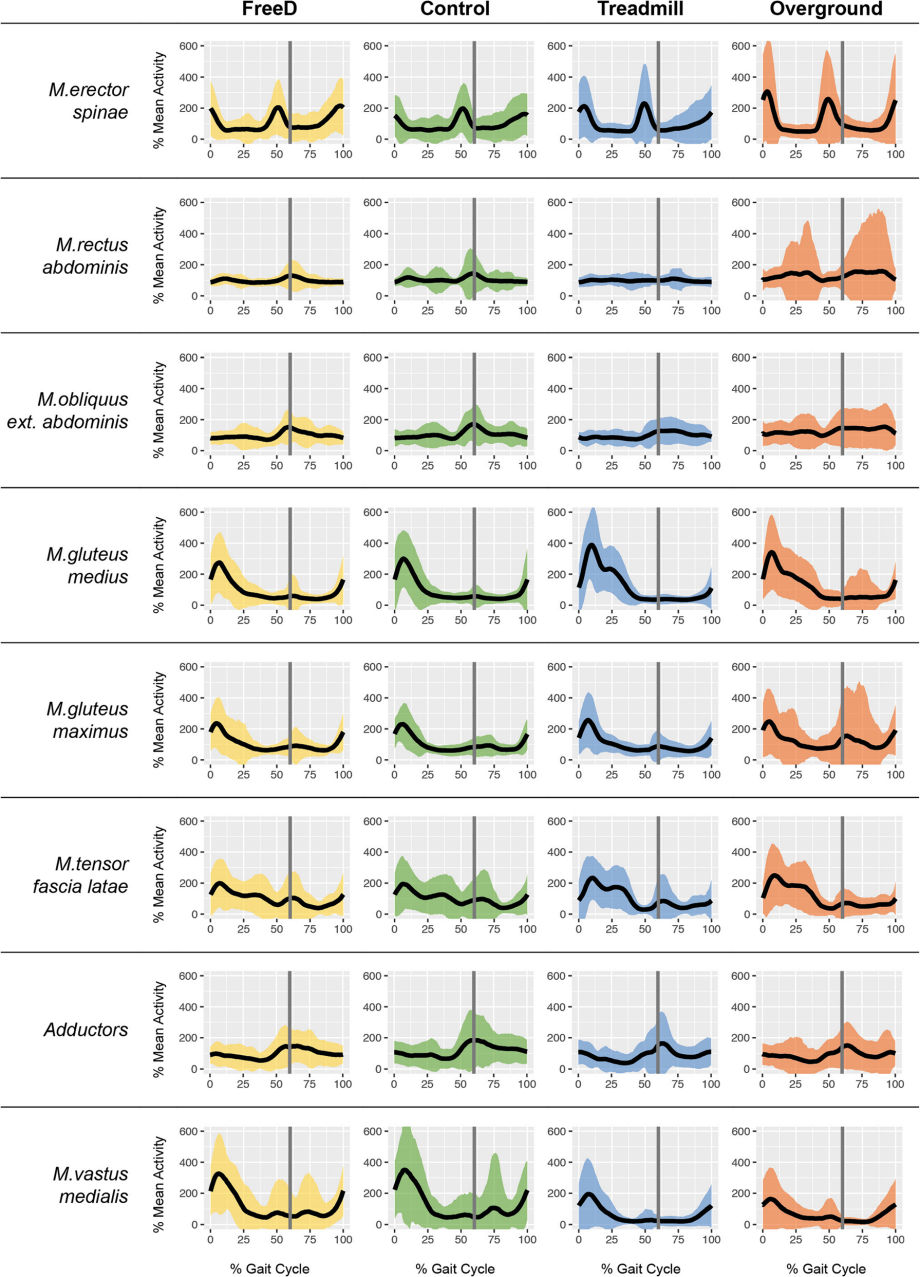
Overview of all averaged sEMG activity normalized to the mean amplitude of Lokomat and treadmill walking. The grey line at 60% of the gait cycle indicates the normalized toe-off. The 95% confidence interval is shown by colored areas. Mean walking speed for all conditions was 3.0 km/h
