# Supplementary material for: The FreeD module for the Lokomat facilitates a physiological movement pattern in healthy people – a proof of concept study
Source: J Neuroeng Rehabil. 2019 Feb 6;16:26. doi: 10.1186/s12984-019-0496-x (PMC6366098; doi:10.1186/s12984-019-0496-x)
Supplement: Supplementary file 1 — Standardized test instructions. (DOCX 16 kb) [file 12984_2019_496_MOESM1_ESM.docx]

Standardized test instructions

Test procedure in general

“During the measurement, we will record the muscle activity and film the reference points of the hip and the chest with a camera. You will walk under 3 different conditions. First in the Lokomat, which will take about 20 minutes. Then, you will walk on the treadmill for approximately 10 minutes and, finally, you will walk on the floor. The instructions during the measurements will be standardized. Try to be very concentrated during this time. Follow my instructions and try to walk as naturally as possible. Please tell me immediately if something is very uncomfortable or painful”

First 2 minutes of Lokomat walking

“Always keep your hands at the same place on the bar, keep your arms and shoulders relaxed. Don't support yourself with your hands.

Always try to walk as actively and normally as possible.

You will now have 2 minutes to walk in the Lokomat until I have adjusted all the final settings.

Would you like to walk faster?”

Trials Lokomat

“We will now measure twice for 10 minutes, with a short interruption in between. Always try to walk as actively and normally as possible. The hands stay always loose on the bars”

Time point 2 minutes: “Try to not become passive” and correction phrases if necessary

Time point 5 minutes: “Keep your hands, arms and shoulders relaxed” and correction phrases if necessary

Time point 8 minutes: “Two minutes left, straighten up again and keep walking actively” and correction phrases if necessary

Trials treadmill

“You will now walk for 10 minutes on the treadmill. Try to walk as normally as possible and don't let the leg straps distract you. Try to keep the position on the treadmill”

Time point 2 minutes: Correction phrases if necessary

Time point 5 minutes: “Keep your hands, arms and shoulders relaxed” and correction phrases if necessary

Time point 8 minutes: “Two minutes left, straighten up again and keep walking actively” and correction phrases if necessary

Correction phrases during Lokomat and treadmill conditions (at time points 2, 5, and 8 minutes, if necessary)

- Straighten up your upper body again, make yourself big”
- “Keep your hands, arms and shoulders relaxed, don't support yourself with your hands”
- “Walk a little bit to the front / back” (position on the treadmill)
- “Control your loading response at heel strike”

Trials overground

“You will now walk this 10 meter walkway several times, at the same speed as in the Lokomat. Try to walk normally”

“Walk the distance between the markings at the given speed. You hear an acoustic signal and you have to adjust your speed so that you are at the next cone at each tone. Stay active and try not to amble. First, we do 3-4 test runs”
